# The viability and appropriateness of using visual methods in end of life research to foreground the experiences of people affected by financial hardship and deprivation

**DOI:** 10.1177/02692163221146590

**Published:** 2023-01-06

**Authors:** Naomi Richards, Sam Quinn, Margaret Mitchell, Emma Carduff, Merryn Gott

**Affiliations:** 1 School of Interdisciplinary Studies, University of Glasgow, Dumfries, UK; 2 Documentary Photographer, Glasgow, UK; 3 Marie Curie Hospice, Glasgow, UK; 4 School of Nursing, The University of Auckland, Auckland, New Zealand

**Keywords:** Photography, socioeconomic factors, palliative care, health equity, data collection, methods, social deprivation, poverty, financial stress

## Abstract

**Background:**

Visual methods have been used extensively in social research to explore people’s experiences of structural disadvantage. This indicates that they may provide a useful research approach to understanding equity-related concerns within palliative care. However, little has been published regarding the use of visual methods with people at the end of life.

**Purpose of the paper:**

In this article we draw on our experiences of using visual methods to illuminate the end of life experiences of people experiencing financial hardship and deprivation in Scotland’s largest city.

**Evidence used to support the information presented:**

We present evidence from the published literature, as well as our own experiences of using visual methods to explore dying at home for people experiencing financial hardship and deprivation. Our analysis draws on two specific visual methods: photovoice and professional photography. Photovoice is a participatory visual method which involves enabling participants to take and discuss their own images and present them to different audiences to try to enact social change. We report our experiences as researchers, as well as those of our participants and recruitment partners.

**Key learning points:**

To successfully use visual methods, researchers need to invest significant time and resource in building a strong rapport with participants. There are also key ethical, practical and representational challenges to consider. A participatory framework should be adopted which ensures agency for participants in terms of image creation and public dissemination. Participants reported value in using visual methods in terms of legacy building and self-representation. Using photovoice (insider’s view) and professional photography (outsider’s view) together offered complementary perspectives, enabling a rich layering of stories and meaning. Our findings indicate visual methods can illuminate aspects of the end of life experience not captured by other research methods.

**What is already known about the topic?**

- Photovoice is a participatory visual method which has an established track record of use in research with people experiencing various forms of structural marginalisation and involves empowering participants to take their own images and represent their strengths and concerns to policy-makers and practitioners;
- There is some evidence of social scientists collaborating with professional photographers in research studies but this visual method is not well documented in the research methods literature;
- Visual methods have rarely been used in palliative and end of life research, likely as a result of sensitivities around representing dying and/or researchers’ lack of experience or confidence in using visual methods or undertaking visual analysis;

**What this paper adds**

- Photovoice is a participatory visual method which is both viable and appropriate for use in end of life research, and specifically with people experiencing structural marginalisation, although it needs to be continually adapted as a result of technical challenges, the physical frailty of participants, and short/unpredictable windows of time to work with participants;
- Professional photography that is undertaken in a way that respects participants’ agency offers an outsider’s view which, when combined with the insider’s view via the photovoice imagery, can generate a rich, multi-layered understanding of a person’s life and struggles;
- The combination of photovoice and professional photography can lead to insights into people’s experiences not accessible through textual or numerical data alone.

**Implications for practice, theory, or policy**

- Visual methods, which show rather than tell audiences about experiences of dying whilst also experiencing financial hardship and deprivation, have the potential to disrupt assumptions about privilege with in palliative and end of life care.
- Images have advantages over textual and numerical accounts in that they can evoke embodied presence and imaginative identification.

## Introduction

Visual research methods are very rarely used in palliative and end of life research, despite being an accepted method in social research for over three decades.^1^ Whilst visual imagery is a pervasive form of communication in Western societies, imagery which specifically shows dying is harder to find, although some does exist.^2–5^ There is a clear lack of images taken by people who are themselves dying, as well as a troubling lack of diversity in terms of *whose* dying experiences are being visually represented. In an occularcentric world, without visual representation these dying experiences do not feature in public discourse and conscience.

In this article, we specifically address the viability and appropriateness of using visual methods in end of life research with people experiencing financial hardship and deprivation. The overarching aim of the research on which this article is based was to use visual methods to examine barriers to, and experiences of, home dying for people experiencing financial hardship and deprivation in the UK.^6–8^ The current UK poverty rate of 22% is predicted to rise significantly in the future as a result of the pandemic, the rising cost of living, and government cuts to public services.^9,10^ People’s socio-economic circumstances can have a profound effect on their end of their life experiences.^7^ Our research was attempting to visually represent such experiences in order to advocate for change.

For Stajduhar,^11^ the ‘typical’ palliative care patient is housed, white, has the support of (biological) family, a strong social network and the financial resources to pay for supplementary care and other costs. The participants in our study did not fit these assumptions. We were, therefore, particularly interested in the potential of visual methods to disrupt assumptions about privilege within palliative and end of life care.

## Background

Visual methods were originally used as a way of trying to objectively document social phenomena via photographs or film. However, over the last 30 years, visual data have come to be understood as a co-construction between researcher and participant, influenced by pre-existing visual and aesthetic tropes.^1^ As such they need to be subjected to in-depth interpretative analysis.^12^ Researcher or participant created visual data encompasses media as diverse as photography, video, drawing, painting, mapping exercises and graphic novel creation. Visual methods can be focused on analysing pre-existing visual representations or creating and then analysing new visual data generated during a study.^1^ Researchers who use visual methods must provide a strong rationale for choosing visual methods over other methods. Of particular interest to researchers who are attracted to using visual methods is the notion that they can reveal insights not accessible by any other method.^1,13^

*Dying in the Margins* was designed as a participatory research project. We chose visual methods which would maximise the agency of participants.^14^ Participatory research encompasses a range of methodological approaches^15^ whose roots can be traced back to social action research and emancipatory philosophy.^16^ What should unite studies which claim a participatory approach is a common ideological position whereby the intention is to rectify the power imbalance between researcher and researched and overcome the ‘symbolic violence’ done to people who are discursively marginalised, for example, as the ‘undeserving poor’.^17^

In this article we report on two visual methods used in the *Dying in the Margins* study: photovoice and professional photography. Photovoice is a participatory research method pioneered in the early 1990s by Wang and Burris^18^ and is designed to: (1) empower participants to document their ‘strengths and concerns’ through the taking of their own photographs; (2) promote critical dialogue and knowledge through discussion of their photographs; and (3) reach policy-makers. Photovoice is rooted in critical pedagogy,^19^ feminist theory and participatory praxis^20^ and there is an established body of literature supporting its use with structurally marginalised groups.^21,22^ The photovoice method is inductive and involves an iterative cycle of research, discussion and action. Participants can see, interpret, and appraise their images as they generate them, discussing the process either collectively in a group or one-on-one with the researcher. These discussions are recorded and form data which are analysed alongside the visual data.^23^ There are reports of benefits for participants in terms of: enhanced self-esteem, confidence and control^24^; the raising of participants’ critical consciousness^25^; and enabling creative expression and meaning-making.^13^

Due to the benefits outlined above, photovoice is an immensely popular method and has even been rather evangelically dubbed ‘the little method that could change the world’.^26^ Certainly, those who use the method can become convinced of its emancipatory power, although more critically-minded scholars suggest implementing it in a ‘committed and critical manner’.^22^ Clearly there are persistent ethical concerns around confidentiality, copyright, interpretation and dissemination. However, as Teti^27^ argues, there exists ‘robust and abundant’ literature on the ethics of using photovoice although the ethical issues are constantly evolving, making it a pointless endeavour to seek out hard and fast rules of application.

There are a number of guides to using photovoice.^18,23^ The method has been used with people with a variety of illnesses^28,29^ and with carers of people at the end of life.^30–32^ However, there are very few examples of use with people who are themselves at the end of life^13,33,34^ and fewer still where the images are published and subjected to visual analysis.^13,34^

The second visual method we discuss is working with a professional photographer. Two of the authors (NR and MG) previously used this method in a study about images of older women.^35^ While visual methods textbooks distinguish between ‘found’, ‘researcher-generated’ and ‘respondent generated’ visual data, there is less guidance available on collaborations between researchers and professional photographers in the generation of new imagery.^36,37^ This is despite there being a long collaborative tradition going back to the 1930s.^38^ The rationale for recruiting a professional photographer to our research team was to convey aspects of our participants’ experiences or feelings that they were not able to capture themselves through photovoice. We also wanted to generate exhibition quality impactful imagery which would draw the attention of policy-makers.

Like photovoice, research involving professional photography in palliative care settings is extremely limited. Examples from perinatal palliative care, where it has been used as a means of legacy building^39^ and to aid the grieving process, involved discussing pre-existing professional imagery with bereaved parents rather than the research team collaborating with a professional photographer to make new images as we did in our project.^40^ More substantive books of images of dying taken by professional photographers do exist, but have not been taken as part of research projects.^2–5^

## Methodological findings

In this section we report on methodological findings from *Dying in the Margins* (data collection 2021–2022) illustrated by visual data and quotes from the study. These insights come from working with eight participants who were: (1) considered by relevant health care providers to be living with serious advanced illness and nearing the end of life and (2) living in areas of relatively high deprivation and/or self-reported as ‘struggling to make ends meet’ or ‘experiencing difficulties getting by on a low income’. We worked closely with recruitment partners (clinical staff, link workers) at two hospices and with so-called ‘deep end’ GP surgeries situated in areas of higher deprivation in Glasgow, Scotland^41^ to identify participants.

Recruited participants were provided with digital cameras (although some preferred to use their phone cameras) and asked to take images of ‘the things and experiences that are important to you, the things you are finding helpful, as well as those you may be worrying about’. This was a longitudinal study and we worked with participants over several months, therefore we chose not to restrict the number of images taken by participants as other studies have done.^13^ All participants were invited to—and indeed agreed to—work with Margaret Mitchell, an award-winning documentary photographer based in Scotland who has an extensive body of work portraying people experiencing structural marginalisation.^42^ Participants were given the choice of whether or not to have their face photographed, but all wanted their face to be shown. Indeed, all participants wanted their real names to be used alongside their images in dissemination of the work. Consent to use both photovoice and professional images was agreed on an image-by-image basis during touchpoints. Copyright of the photovoice images belongs to the project, while Margaret Mitchell retains copyright of her images.

### Practicing visual methods

**Figure 1.**
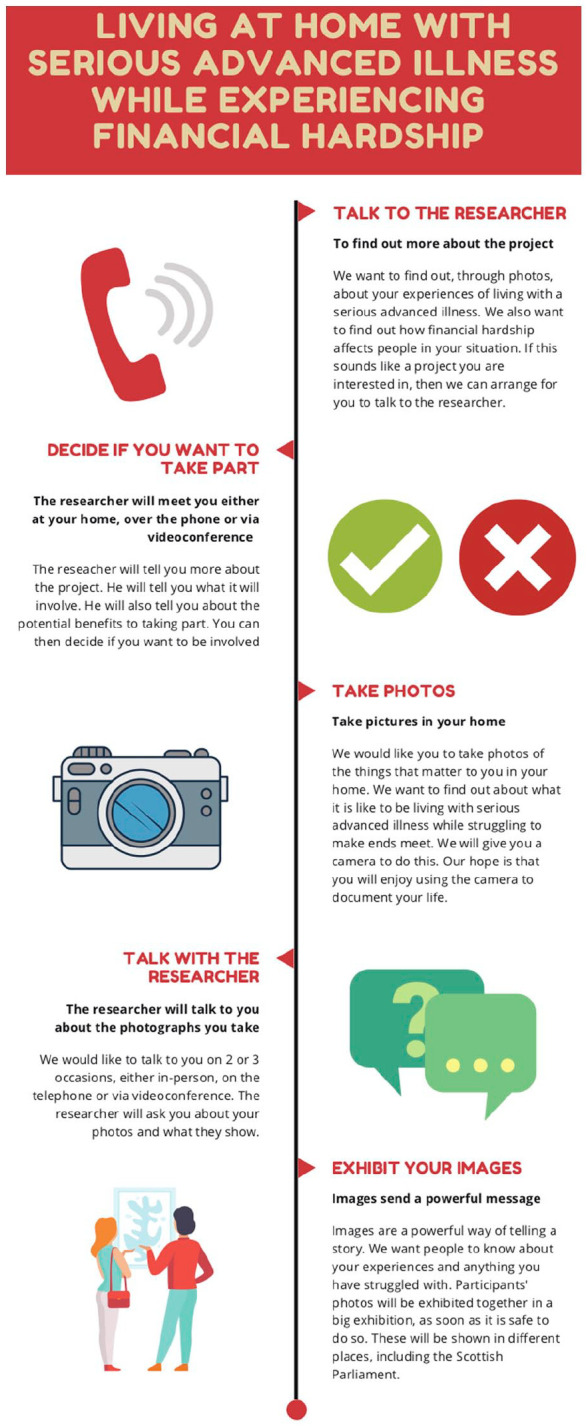
Visual flowchart showing the method used with potential participants and recruiters. ©Dying in the Margins 2021 all rights reserved

Our use of visual methods with people at the end of life coincided with the COVID-19 pandemic which inevitably compounded many of the practical challenges faced. The first challenge was the necessary translation process whereby the visual methods were carefully explained to recruitment partners unfamiliar with their purpose and provenance. We provided our recruitment partners with a script and visual aids for use with potential participants (Figure 1) but we found that the best way to convince them of the appropriateness of our methods was to share participant and photographer produced images *as they emerged*. From the perspective of the research team, the images undoubtedly became our strongest recruitment tool. Recruitment partners also witnessed the beneficial effects on participants of taking part which resulted in more referrals to the project (see Section 2 for more details about the beneficial effects).

**Figure 2.**
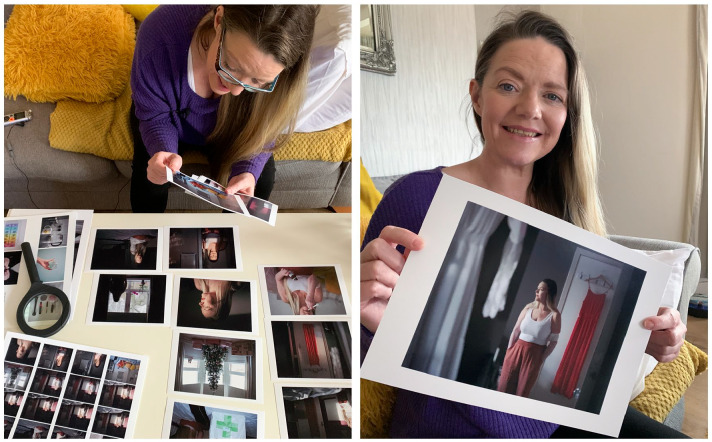
Photographer Margaret Mitchell returns to show photographs to a participant. ©Margaret Mitchell 2022 all rights reserved

Once recruited, regular telephone or email check-ins with participants were required to avoid attrition, something which is documented in other (non-visual methods) studies with structurally marginalised populations.^43,44^ Participants’ involvement fluctuated depending on what was happening in their life. For example, a participant went into hospital and we did not hear from them for a month, or another participant’s project camera disappeared with accusations levelled at friends. We found regular communication and the steady building of rapport, with both the researcher and Margaret the photographer, was key to combating waning involvement. We emphasised to participants that people would be interested in what they had to share about their lives. Seeing the professional images in tangible form quickly after they were taken also helped them to feel invested in the study and reduce the risk of attrition (Figure 2).

For participants with computer access and a degree of computer literacy, we sent them weekly ‘drop off’ requests where they could securely upload their photovoice images. However, the majority of our participants did not have a computer or the technical capability to do this. In-person touchpoints were used to download images from cameras but were difficult to arrange during the pandemic. This led to concerns that people would die or would drop out and data would be lost. When safe to do so, in-person touchpoints were arranged approximately once a month. Free-flowing discussions were had about what the images showed, which images participants liked best, and any they did not want used or exhibited. Participants also discussed what they had wanted to capture but had not been able to.

All participants had some degree of frailty which affected their ability and inclination to take photographs. One participant had full body muscle atrophy which required us to identify alternative ways for him to take photographs. Others had waning energies as a result of palliative chemotherapy or because they were actively dying. In designing the study, we had envisaged that family carers could facilitate the image taking, and even become the primary photographer in the final weeks of life. In reality, recruiting and then staying in contact (remotely) with both the participant and their carer was challenging as both had to be equally committed to the project. Some of our participants were single and their carers were friends, parents or children. Carers were also struggling with financial insecurity themselves and not always easily contactable. Instead of relying on carers, our main strategy for facilitating participant’s involvement at the very end of life, was to invite them to work with documentary photographer, Margaret Mitchell. The effects of this strategy are detailed in the next section.

Our project confirmed what is well established in the literature- that reaching and enabling the contributions of the most disenfranchised populations requires significant researcher time and labour, as well as trust.^45^ We were engaged in the ‘art of the possible’ and it wasn’t always possible to stick dogmatically to pre-determined protocols. As emphasised by Teti,^27^ facilitating participation required ongoing consideration of ethics and adaptation of methods at every stage.

### The promise of visual methods

**Figure 3.**
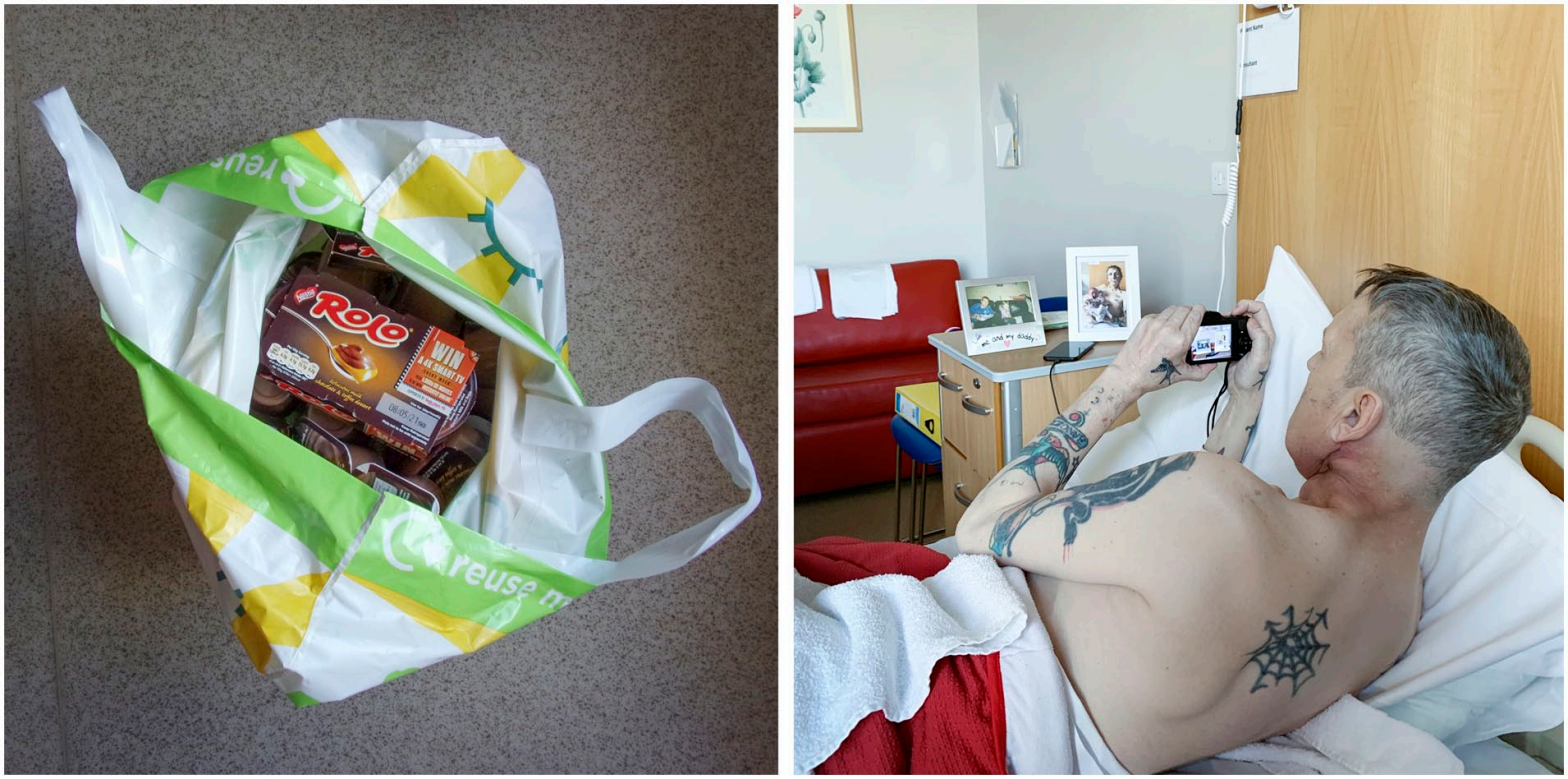
A participant shown taking photovoice images of a bag of chocolate puddings—the only thing he was able to eat—from his hospice bed. Left hand photograph ©Dying in the Margins 2021; right hand photograph ©Margaret Mitchell 2021 all rights reserved

Upon seeing the visual data from our first participant in April 2021, theoretical support for our chosen methods consolidated into a confirmed conviction about their power and potential. Our first participant, Andy, had experienced what is known in policy terms as ‘deep and persistent’ poverty.^9^ He was a hospice inpatient with no option to return home since his flat had been broken into and boarded up by the council. In the month he was involved in the study, Andy managed only a few photovoice images, all taken from his hospice bed (e.g. Figure 3). His physical frailty prohibited him taking more, but he was able to work closely with Margaret on three occasions in the last weeks of his life. This shows the value of the combination of the two methods when working specifically with participants who are near the end of their life. Box 1 details Margaret’s process in her own words as she bore witness to the validation which Andy experienced seeing himself represented in her portraits.

**Box 1.** Documentary photographer’s practice explained.

**Figure 4.**
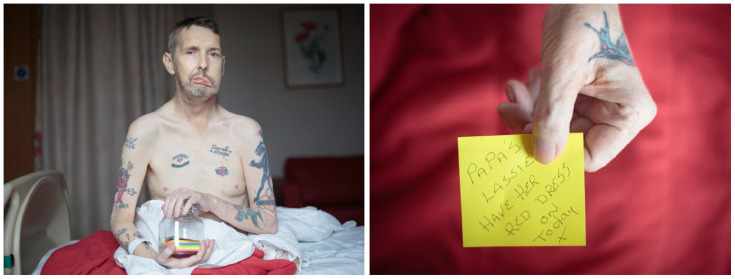
Participant Andy holds a jar of notes he wrote for his granddaughter in a portrait taken by photographer Margaret Mitchell. ©Margaret Mitchell 2021 all rights reserved

**Professional Documentary photographer’s practice (by Margaret Mitchell)**
Being a documentary and portrait photographer is a privileged position where I’m invited into people’s lives to represent part of their story. My work comes from a place of observation, engagement and listening, leading to the final images. In coming onto the *Dying in the Margins* project, I brought with me a methodology grounded in visual storytelling, empathy and respect. Within my documentary practice, I make repeat visits to those I photograph which allows a multifaceted picture to be built up facilitating both nuance and depth. I always return with prints and seek participants’ opinion, ensuring approval at every step (see Figure 2).
The role of a photographer is in some ways that of translator: that we see a scene, a circumstance and work on how to represent that to a wider public. But most importantly for me, we must keep the person photographed as the priority in every image and ensure that their agency is upheld. Having a working process that emphasises explanation, dialogue and encourages feedback, helps to nurture a respectful and equal relationship. In my work, I have always rejected facile interpretations of situations that are complex. People living in disadvantage are frequently portrayed by visual stereotypes and sensationalism with the intricate realities of their situations often ignored.
The ethics of photographic representation runs deep through all my work, but nowhere have I felt this more keenly than in working with people at the end of their lives. When I met Andy, his illness affected his ability to talk with ease and using visual methods was advantageous.^46^ I noticed his jar of notes where he was writing messages to leave behind for his baby granddaughter (see Figure 4). This very personal aspect of Andy’s story was so tender and poignant and led to the portrait of him holding the jar. I photographed Andy as I found him; his life history, in part, written through the tattoos on his body. Andy’s reaction on seeing the first set of images was that he wanted them bigger, printed large. People like Andy often go through life being judged, ignored, and disregarded due to a life lived in disadvantage. What I hope we achieved on this project is a recognition of not only Andy as an individual but also that unequal starting points in life are often reflected at end of life too.

The pride which Andy expressed when he saw Margaret’s images of him shows the power of professional images to capture an aspect or essence of a person which may otherwise be inexpressible. Another participant, Marie, confirmed this when she said that Margaret was ‘trying to capture the essence of how I’m feeling . . . the atmospherics’. Margaret’s images, through their ‘atmospherics’, evoked the embodied presence of the individuals and, as we will discuss in future articles, promoted imaginative identification when viewed by others.

**Figure 5.**
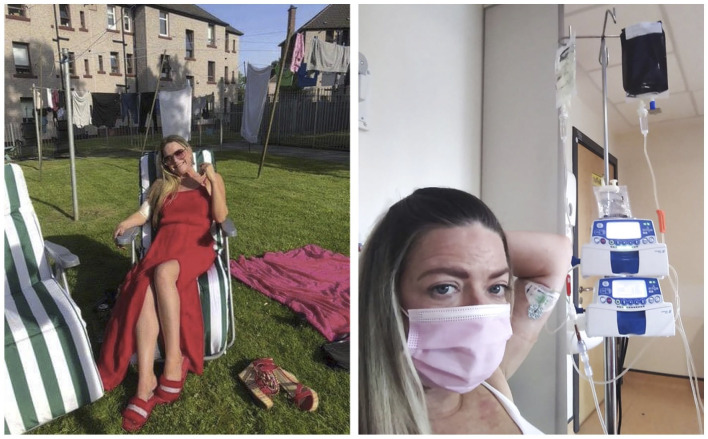
Marie’s photovoice images. ©Dying in the Margins 2021 all rights reserved

Both the professional photographs and the photovoice images contributed to legacy building and meaning-making for participants. For Marie, it was the photovoice which gave her the opportunity to legacy build, documenting her life to be shared with her children after her death (e.g. Figure 5):

I do have [the photos] saved for my daughter and so when the time comes, she can have a copy and she can look back. I think that’s been beneficial to me, almost like a diary.

For another participant, legacy came in the form of a portrait image being used at the hospice’s memorial service and printed copies supplied for his close friends. The project was also mentioned in his funeral eulogy.

**Figure 6.**
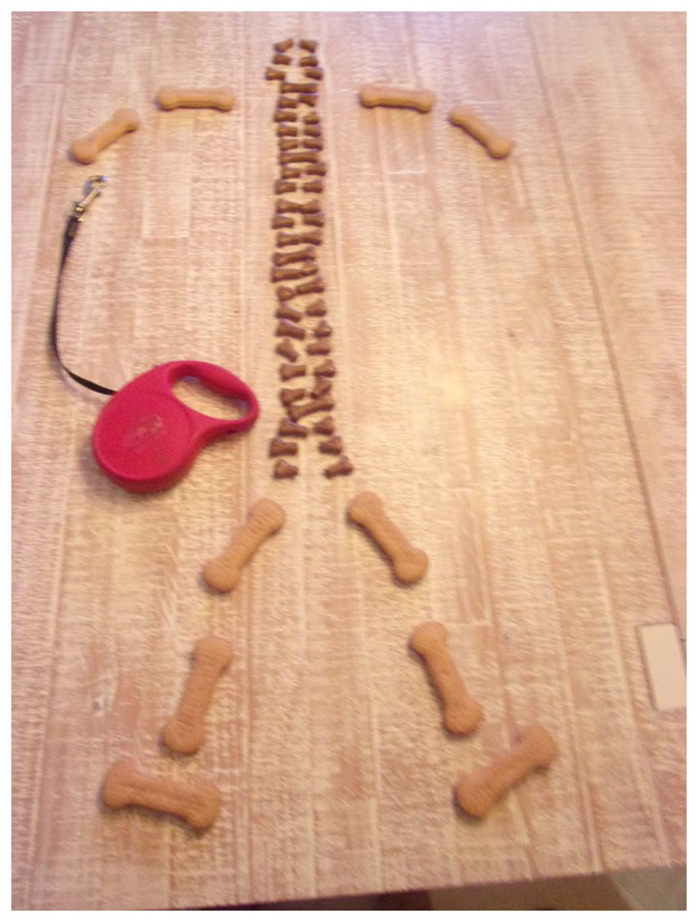
A participant’s photovoice image representing how her arthritis and osteoporosis prevent her from walking her dog, one of her favourite pastimes. ©Dying in the Margins 2021 all rights reserved

Some participants found the photovoice method presented an opportunity for creativity and to express experiences and perspectives which, in an interview or focus group, they might otherwise have struggled to articulate (e.g. Figure 6). As one participant expressed it:

I hope by taking part in this study that I will be a voice for people like me who are struggling.

And at another touchpoint:

We all find coping strategies [. . .] the estate is apparently being demolished, but before it is I’m going to take photos of it [. . .] I try and have these projects on the go, something to look forward to and keep my head above.

While participants, like Andy, who were close to death only managed a few images, other participants took hundreds over several months showing their investment in the method. This level of engagement provides immensely rich, in-depth data.

**Figure 7.**
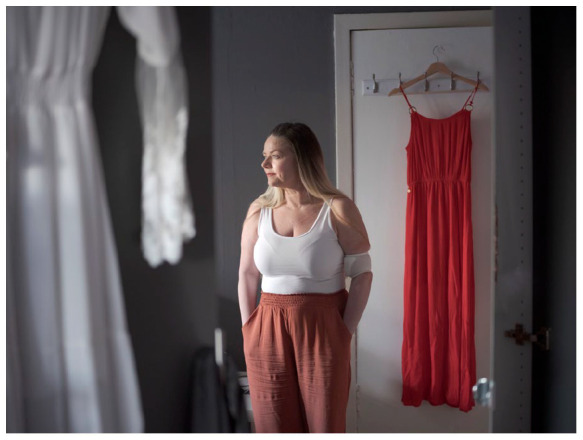
A portrait of Marie taken by documentary photographer Margaret Mitchell. ©Margaret Mitchell 2021 all rights reserved

The two visual methods combined to allow both insider and outsider perspectives and this has expanded our understanding of participants’ circumstances. For example, while Marie’s photovoice revealed how she *wanted* to be seen (young, glamorous, coping) Margaret’s photos added nuance to her situation, incorporating reflection and poignancy (e.g. Figure 7). While some participants were taken aback seeing portraits of themselves up closebecause they felt conscious of their altered physicality, this prompted critical reflection on their situation and on time left. In all cases, Margaret’s images revealed aspects of people’s lives which the insider view/photovoice imagery did not, and vice-versa. The two types of images have different qualities and can be juxtaposed or layered to enhance meaning or emotional identification beyond *just* photovoice or *just* professional imagery.

### Potential pitfalls of visual methods

The main pitfall we encountered utilising visual methods relates to sensitivities around photographing experiences of financial hardship and deprivation rather than experiences of dying. The research team had more collective knowledge about the sensitivities of discussing and representing dying and we underestimated how stigma and shame formed the hidden emotional context when discussing and representing poverty. Within healthcare there are various initiatives to try to promote open conversations about dying.^47^ However, there is less public discussion and fewer initiatives about how to talk sensitively to people about their experiences of poverty and material hardship. This has led some to argue for training in ‘structural competency’ for healthcare professionals.^48^ On several occasions, those we were trying to bring on board as recruitment partners referred to the idea that taking photographs could be exposing and shaming for people. To quote one nurse’s perception of the project: ‘you’ve got a shit life, so I want you to take pictures of it’. Such comments are troubling because they reveal the judgement which people can perceive in identifying people who are experiencing financial hardship.

Our participatory approach was vital here. Our intention was to give people the means and the platform to make visible their own struggles and strategies *in a way that was culturally appropriate to them*. Margaret also brought long-standing expertise in photographic representations of class and social inequality.^49^ In a neoliberal context where politicians and the media deny or misrepresent the poverty and hardship which some people experience, there is a political need to rectify such an impression and make visible the full spectrum of material circumstances which people face.^50^ To quote the photography book *Invisible Britain*:

What the eye does not see is what they don’t want you to see, or think over, or act on.^51^

However, as Switzer^52^ argues, even photographs produced in a participatory project can unintentionally stigmatise people and reinforce rather than disrupt negative stereotypes. There is significant responsibility which we carry as researchers and artists in representing people’s lives in a way which narrates with care and respect. But crucially we also did not want to hide people’s struggles and material hardship. Debates about representation have been a central feature of this study and future articles will focus on the public display and reception of the images. Our main argument here is that there are heightened sensitivities involved when visually representing both dying *and* financial hardship. In order to retain confidence in using these methods, respect for the agency of each participant was fundamental: that their choice to take part, take photographs, be photographed, and to be seen in public display was respected. Undoubtedly, visual methods are not acceptable to everyone. A number of potential participants declined to take part specifically because it was an image-based study, meaning stories were lost. However, the visual data from this study is leading to insights not accessible, and effects on participants not achievable, through textual or numerical data alone.

## Conclusion

This article has outlined the practical, ethical, and representational challenges involved in using photovoice and professional photography specifically with people who are dying within the context of financial hardship and deprivation. The two methods can confer benefits on participants for different reasons. Photovoice enabled participants to explore their own creativity and express their vision of what was important to them and what they wanted to change. Working with a sensitive and experienced professional photographer helped to make them feel seen and to be represented with strength in a way that enhanced self-reflection. Both methods contributed to legacy building for our participants.

Throughout the study we have also been made aware of the significant responsibility that accompanies visually representing the lives of people who are structurally marginalised, especially in a neoliberal context where the existence of poverty is either denied or deemed the responsibility of the individual.^50^ However, any risks associated with displaying the images has to be balanced with our participants’ right to choose to have their voices amplified, to create legacy and exert control over their own representation. Critically, when used together, photovoice and professional photography were complementary methods, representing both the insider (emic) and outsider (etic) views of the life lived and enabling the rich layering of stories and meaning. The lack of representation of people who are dying whilst worrying about how they will pay their bills, living in basement or third floor flats unable to get outside for fresh air, who spend their last weeks of life trying to convince their social housing provider to fix lights, mould, and damp, all means that such circumstances, experienced by our participants and represented in project photographs, are not taken into consideration when designing services and policy. Once displayed side-by-side, the photovoice and professional images with their complementary qualities and perspectives will contribute to efforts to displace the dominant story about the ‘typical’ palliative care patient.

## References

[1] Banks M . Using visual data in qualitative research. 2nd ed. London: SAGE Publications Ltd, 2018.

[2] Andre D Brookman P Livingston J . Hospice: a photographic inquiry. 1st ed. Boston, MA: Bulfinch Press, Little, Brown and Company, Corcoran Gallery of Art, 1996.

[3] Ennis H . Reveries: photography & mortality. Canberra: National Portrait Gallery, 2007.

[4] Jury D Jury M . Gramp: a man ages and dies. The extraordinal record of one family’s encounter with the reality of dying. New York, NY: Grossman, 1978.

[5] Lakotta B Schels W . Noch mal leben vor dem Tod: Wenn Menschen sterben. München: Deutsche Verlags-Anstalt, 2004.

[6] UK Research & Innovation. Dying in the Margins: uncovering the reasons for unequal access to home dying for the socio-economically deprived, https://gtr.ukri.org/projects?ref=ES%2FS014373%2F1 (2019). (accessed 22 December 2022).

[7] Rowley J Richards N Carduff E , et al. The impact of poverty and deprivation at the end of life: a critical review. Palliat Care Soc Pract 2021; 15: 26323524211033873. 34541536 10.1177/26323524211033873 PMC8442481

[8] Richards N . The equity turn in palliative and end of life care research: lessons from the poverty literature. Sociol Compass 2022; 16: e12969.

[9] Social Metrics Commission. Measuring poverty 2020: A Report of the Social Metrics Commission, July 2020.

[10] Marmot M . Lower taxes or greater health equity. Lancet 2022; 400(10349): 352–353. 35908567 10.1016/S0140-6736(22)01392-7

[11] Stajduhar KI . Provocations on privilege in palliative care: are we meeting our core mandate? Prog Palliat Care 2020; 28(2): 89–93.

[12] Riessman CK . Narrative methods for the human sciences. London: SAGE Publishing, 2008.

[13] Tishelman C Lindqvist O Hajdarevic S , et al. Beyond the visual and verbal: Using participant-produced photographs in research on the surroundings for care at the end-of-life. Soc Sci Med 2016; 168: 120–129. 27643846 10.1016/j.socscimed.2016.09.012

[14] Mitchell C Lange ND Moletsane R . Participatory visual methodologies: Social Change, community and policy. London: SAGE Publications Ltd, 2018.

[15] Bergold J Thomas S . Participatory research methods: a methodological approach in motion. Hist Soc Res 2012; 13(1): 191–222.

[16] Macaulay AC . Participatory research: what is the history? Has the purpose changed? Fam Pract 2017; 34(3): 256–258. 27993909 10.1093/fampra/cmw117

[17] Bourdieu P Wacquant LJD . An invitation to reflexive sociology. 1st ed. Chicago, IL: University of Chicago Press, 1992.

[18] Wang C Burris MA . Photovoice: concept, methodology, and use for participatory needs assessment. Health Educ Behav 1997; 24(3): 369–387. 9158980 10.1177/109019819702400309

[19] Freire P . Pedagogy of the oppressed. London: Bloomsbury, 1970.

[20] Wang CC . Photovoice: a participatory action research strategy applied to women’s health. J Womens Health 1999; 8(2): 185–192. 10100132 10.1089/jwh.1999.8.185

[21] Loignon C Dupéré S Bush P , et al. Using photovoice to reflect on poverty and address social inequalities among primary care teams. Action Res 2020; 8: 147675032090590.

[22] Liebenberg L . Thinking critically about photovoice: achieving empowerment and social change. Int J Qual Methods 2018; 17(1): 1–9.

[23] Milne EJ Muir R . Photovoice: a critical introduction. In: Pauwels L Mannay D (eds) The SAGE handbook of visual research methods. London: SAGE Publications, Inc, 2020, pp.282–297.

[24] Teti M Pichon L Kabel A , et al. Taking Pictures to take control: photovoice as a tool to facilitate empowerment among poor and racial/ethnic minority women with HIV. J Assoc Nurses AIDS Care 2013; 24(6): 539–553. 24064314 10.1016/j.jana.2013.05.001 PMC3883445

[25] Carlson ED Engebretson J Chamberlain RM . Photovoice as a social process of critical consciousness. Qual Health Res 2006; 16(6): 836–852. 16760539 10.1177/1049732306287525

[26] Evans-Agnew RA Strack RW . Photovoice: the little method that could change the world. Health Promot Pract 2022; 23(2): 201–204. 35285329 10.1177/15248399211069151

[27] Teti M . The murky ethics of visual qualitative methods: picturing a clear path forward. Int J Qual Methods 2019; 18: 160940691988481.

[28] Balmer C Griffiths F Dunn J . A ‘new normal’: exploring the disruption of a poor prognostic cancer diagnosis using interviews and participant-produced photographs. Health 2015; 19(5): 451–472. 25323052 10.1177/1363459314554319

[29] Thompson NC Hunter EE Murray L , et al. The experience of living with chronic mental illness: a photovoice study. Perspect Psychiatr Care 2008; 44(1): 14–24. 18177274 10.1111/j.1744-6163.2008.00143.x

[30] Horsfall D Yardley A Leonard R , et al. End of life at home: co-creating an ecology of care. Penrith, NSW: Western Sydney University, 2015.

[31] Hajradinovic Y Tishelman C Lindqvist O , et al. Family members’ experiences of the end-of-life care environments in acute care settings - a photo-elicitation study. Int J Qual Stud Health Well Being 2018; 13(1): 1511767. 10.1080/17482631.2018.1511767 PMC6127834 30176152

[32] Campbell LM . Experiences of nurses practising home-based palliative care in a rural South African setting. Int J Palliat Nurs 2011; 17(12): 593–598. 22240741 10.12968/ijpn.2011.17.12.593

[33] Bates MJ Ardrey J Mphwatiwa T , et al. Enhanced patient research participation: a photovoice study in Blantyre Malawi. BMJ Support Palliat Care 2018; 8(2): 171–174. 10.1136/bmjspcare-2017-001439 PMC5969331 29420196

[34] Moore A Carter B Hunt A , et al. ‘I am closer to this place’–space, place and notions of home in lived experiences of hospice day care. Health Place 2013; 19: 151–158. 23247424 10.1016/j.healthplace.2012.11.002

[35] Richards N Warren L Gott M . The challenge of creating ‘alternative’ images of ageing: Lessons from a project with older women. J Aging Stud 2012; 26(1): 65–78.

[36] Rose G . Visual methodologies: an introduction to researching with visual materials. 4th ed. London: Sage Publications Ltd., 2016.

[37] Prosser J Loxley A . Introducing visual methods. ESRC National Centre for Research Methods Review Paper. October 2008. https://eprints.ncrm.ac.uk/id/eprint/420/1/MethodsReviewPaperNCRM-010.pdf. (accessed 22 December 2022).

[38] Goggans J . California on the breadlines: Dorothea Lange, Paul Taylor, and the making of a new deal narrative. Berkeley, CA: University of California Press, 2010.

[39] Boles JC Jones MT . Legacy perceptions and interventions for adults and children receiving palliative care: a systematic review. Palliat Med 2021; 35(3): 529–551. 33487090 10.1177/0269216321989565

[40] Martel SL Ives-Baine L . “Most prized possessions”: photography as living relationships within the end-of-life care of newborns. Illn Crisis Loss 2014; 22(4): 311–332.

[41] University of Glasgow. The Scottish deep end project, https://www.gla.ac.uk/researchinstitutes/healthwellbeing/research/generalpractice/deepend/ (2022). (accessed 22 December 2022).

[42] Mitchell M . Margaret Mitchell. https://margaretmitchell.co.uk (2022). (accessed 22 December 2022).

[43] Stajduhar KI Mollison A Giesbrecht M , et al. “Just too busy living in the moment and surviving”: barriers to accessing health care for structurally vulnerable populations at end-of-life. BMC Palliat Care 2019; 18(1): 11. 30684959 10.1186/s12904-019-0396-7 PMC6348076

[44] Song J Wall MM Ratner ER , et al. Engaging homeless persons in end of life preparations. J Gen Intern Med 2008; 23(12): 2031–2036; quiz 2037. 18800207 10.1007/s11606-008-0771-1 PMC2596520

[45] Kapilashrami A Marsden S . Examining intersectional inequalities in access to health (enabling) resources in disadvantaged communities in Scotland: advancing the participatory paradigm. Int J Equity Health 2018; 17(1): 83. 30244682 10.1186/s12939-018-0797-x PMC6151920

[46] Balmer C Griffiths F Dunn J . A review of the issues and challenges involved in using participant-produced photographsin nursing research. J Adv Nurs 2015; 71(7): 1726–1737. 25678407 10.1111/jan.12627

[47] Bailey SJ Cogle K . Talking about dying: How to begin honest conversations about what lies ahead. Royal College of Physicians, London, 2018.

[48] Metzl JM Hansen H . Structural competency: theorizing a new medical engagement with stigma and inequality. Soc Sci Med 2014; 103: 126–133. 24507917 10.1016/j.socscimed.2013.06.032 PMC4269606

[49] Mitchell M . Passage. Liverpool: Bluecoat Press, 2021.

[50] Shildrick T . Poverty propaganda exploring the myths. Bristol: Policy Press, 2018.

[51] Sng P . (ed.) Invisible Britain: portraits of hope and resilience. Bristol: Policy Press, 2018.

[52] Switzer S . What’s in an image?: towards a critical and interdisciplinary reading of participatory visual methods. In: Capous-Desyellas M Morgaine K (eds) Creating social change through creativity. Cham: Springer International Publishing, 2018, pp.189–207.

